# Effectiveness of Hyaluronic Acid Gel Injection with and without PRGF for Management of Interdental Papillary Loss: A Randomized Clinical Trial

**DOI:** 10.3390/jfb14020114

**Published:** 2023-02-18

**Authors:** Aishwarya Bal, Saurav Panda, Rinkee Mohanty, Anurag Satpathy, Rashmita Nayak, Margherita Tumedei, Francesca Argenta, Gianluca Colapinto, Massimo Del Fabbro, Marco Annunziata

**Affiliations:** 1 Department of Periodontics, Institute of Dental Sciences, Siksha O Anusandhan University, Bhubaneswar 751003, India; 2 Department of Biomedical, Surgical and Dental Sciences, Università degli Studi di Milano, 20122 Milan, Italy; 3 Fondazione Ca’Granda IRCCS Ospedale Maggiore Policlinico, 20122 Milan, Italy; 4 Chief Medical Officer Oral Med Care srl, 70032 Bitonto, Italy; 5 Multidisciplinary Department of Medical-Surgical and Dental Specialties, University of Campania “Luigi Vanvitelli”, 80138 Naples, Italy

**Keywords:** deficient interdental papilla, injectable hyaluronic acid gel, PRGF

## Abstract

Background: To evaluate the effectiveness of hyaluronic acid (HA) gel injection with and without plasma rich in growth factors (PRGF) for the management of interdental papillary loss. Methods: A single blinded randomized clinical trial was carried out on 21 subjects with 34 sites. Patients within the age group 18–45 years who had Class I and II papillary recession in the maxillary anterior region were selected. The sites involved were randomly assigned to Group HA alone and Group HA + PRGF. The patients were recalled 4 weeks after receiving supragingival and subgingival instrumentation. HA or HA + PRGF was injected into the defective papilla at baseline and at 3 and 6 weeks. Image based measurements of Papillary Width (PW), Papillary Deficient Height (PDH), Deficient Area (DA), Deficient Volume (DV) were registered at baseline, 3 weeks, 6 weeks and 12 weeks. A vernier caliper was used to measure the papillary depth in the impression made using additional silicone impression material pre- and post-intervention. Results: There was a significant improvement in the within-group comparison of PW, PDH, DA and DV in both the groups. Group HA + PRGF showed significantly greater improvement in comparison to Group HA alone in terms of PDH, DA and DV at 6 and 12 weeks. Conclusions: Even though HA gel has already been established as a promising injectable agent in the minimally invasive treatment of interdental papillary deficiency, PRGF may also have a significant adjuvant effect when used along with HA. Further clinical studies with longer follow up duration, larger sample size and standardization of the tooth shape are required for a better understanding of the adjuvant effect of PRGF when used along with HA.

## 1. Introduction

The interdental papilla is the gingival tissue that completely fills the interdental space, with the tip close to the contact point; the morphology of the teeth determines the volume and shape of the interdental papilla. Gingival embrasure is the situation where the papilla is missing, cervical to the interproximal contact. It is defined as “open” (“black triangle”) if the space is partially filled by the gingival tissue due to a partial or complete loss of interdental papilla [1]. The black triangle in the anterior region affects the aesthetics of the smile and generates different forms of discomfort, such as difficulty in speaking and impaction of food between the teeth. Kokich et al. considered a gingival black space greater than 3 mm as an evident aesthetic problem for the clinicians and the patients [2]. Gingival black spaces in the past were considered one of the main unaesthetic problems apart from caries and crown margins [3].

The prevalence of open gingival embrasures is rather high in the adult population, with 38% of the cases being represented by the patients undergoing orthodontic treatment [4]. Open embrasures prevalence is 67% of the population older than 20 years and 18% of the population under 20 years of age. Open embrasures are further associated with periodontal disease, periodontal surgery, and orthognathic surgery, which explains their prevalence in adults. Gingival embrasures etiology is multifactorial and the main causes are the dimensional changes of papilla during orthodontic alignment, loss of periodontal attachment resulting in recession, loss of height of the alveolar bone relative to interproximal contact, and poor crown shape reconstruction [5]. It is important to understand the etiology of such a condition, so as to design a patient-oriented treatment plan. Treatment approaches may therefore vary from causal therapy, to eliminate and control the main aetiologic/risk factors through oral hygiene instructions, to papillary mechanical stimulation and non-surgical periodontal treatment followed by re-evaluation, to, lastly, in the case of severe defects, surgical treatment [6,7,8]. One of the most common surgical treatments for interdental papilla loss involves the usage of connective tissue grafts. In spite of its effectiveness, this procedure is cumbersome and invasive and requires strict oral hygiene protocols. This significantly propelled the need for non-surgical and minimally invasive approaches for the augmentation of the interproximal tissue.

Among the nonsurgical approaches proposed, injecting a dermal filler such as hyaluronic acid (HA) became the most popular. HA is a natural linear polysaccharide of the synovial fluid, of the extracellular matrix of connective tissue, and of other tissues [3,4,5,6]. HA preparations obtained from bacterial or animal sources are used as fillers, and their clinical effect has a typical duration of 6 to 12 months [9]. Injecting HA into the connective tissue has been proposed to solve the issue of interdental papilla recession, by promoting the migration of fibroblasts and fibrogenesis [3,10]. Hitherto, a handful of studies have described the effect of injecting HA as a filler for lost interdental papilla reconstruction, and most previous studies were case series with a relatively small sample size [11,12,13,14,15,16,17,18,19]. All these studies concluded that the use of HA to treat deficient interdental papilla results in significant improvement, and therefore it appears as a promising therapy to address patients’ aesthetic concerns.

Autologous platelet concentrates have been subjected to considerable scientific investigation in the last 15 years, and are currently been widely employed in surgical and nonsurgical techniques in periodontics [20,21,22]. Some surgical techniques of managing the lost interdental space and gingival recession defects so far have used platelet-rich fibrin (PRF) as a filler along with connective tissue grafts [23,24,25]. Using the standard protocol for PRF, a clot is obtained after the centrifugation, which is not feasible to be injected. Only recently a special protocol for obtaining an injectable form of PRF (i-PRF) was developed, which still requires validation from the scientific literature [20]. Plasma rich in growth factors (PRGF) is a second-generation system. As compared to first-generation platelet concentrates, PRGF requires less blood; is cheaper, easier to use, and safer; and the preparation time is faster [26,27,28]. PRGF can be used both as a clot, for topical application, and in the liquid/gel form for injections or microinjections into tissues. For example, there is evidence of its effectiveness as a dermal filler in facial rejuvenation treatment [25]. Preliminary in vitro and in vivo studies reported that the treatment with PRGF may have a synergistic effect with hyaluronic acid, improving the biological features of the latter [25,26]. However, scarce evidence has been published where platelet concentrates have been injected as a dermal filler in the interproximal tissue to manage papillary loss alone or in combination with HA.

Hence, the present investigation, the first of its kind, aimed to compare a combination of injectable PRGF and hyaluronic acid for the management of interdental papillary loss, as compared to HA alone. The null hypothesis was that the adjunct of PRGF provided no additional benefit to the treatment with HA.

## 2. Materials and Methods

The current investigation was a single blind randomized clinical trial with a mixed parallel arm/split mouth design, conducted among the patients who visited the Bhubaneswar Dental Hospital from December 2019 to November 2021. This study was performed in agreement with the ethical guidelines of the Helsinki Declaration of 1975, as revised in 2000. The protocol of the study was approved by the Research Ethics Commission of the Siksha ‘O’ Anusandhan (deemed to be a university), Bhubaneswar (DRI/IMS.SH/SOA/2021/152). All the patients enrolled signed a written informed consent form and were aware of the techniques and the complications associated with the procedure employed.

### 2.1. Selection Criteria

The inclusion criteria were: Patients with esthetic concern; complaining of food lodgment in the anterior embrasure; with Class I and Class II papillary loss (Nordland and Tarnow classification, 1982); having adequate width of attached gingiva; within age group of 18–45 years; with plaque index <1 (Turesky, Gilmore and Glickman Modification of Quigley Hein 1970); and gingival index <1 at the involved sites (Loe and Silness 1967). The sites with a distance ≤7 mm from the interdental contact point to the interproximal bone crest and a probing depth of ≤4 mm at the defective papillary sites were considered for inclusion. For split mouth cases, the two interdental spaces had to not be adjacent, to avoid potential paracrine effects of the substances investigated. Patients excluded from the study were those that received radiotherapy, chemotherapy, immunosuppressive treatments, systemic corticosteroids and/or anticoagulants the 30 days prior to intervention; having known history of allergy, systemic or blood borne diseases; prolonged treatment with non-steroidal anti-inflammatory drugs (NSAIDs) or similar medications; smokers; lactating or pregnant females; presence of composite and prosthetic restoration in maxillary anterior region; undergoing orthodontic treatment; having high frenum attachment; having midline diastema; and having any inability to take part in the investigation and comply with the required follow-up procedures. Sites with Nordland and Tarnow Class III papillary loss, sites with underlining intraosseous defects and implant sites were also excluded.

### 2.2. Sample Size Calculation

A sample size calculation was performed similarly to previously published work, which showed a significant difference in the improvement of papillary height (*p* = 0.047) between the control and test group for an effect size of 1.07, α error of 0.05 and power of 0.8. Fifteen sites were needed in the test and control group. Accounting for a possible 10% loss to follow up, a total sample size of at least 34 sites was planned to be recruited.

### 2.3. Randomization and Clinical Procedure

The sites were randomized by the toss or flip of a coin and allocated in two groups by an independent allocator to receive either HA alone or PRGF in adjunct to HA. Allocation concealment was obtained through opaque, sealed and consecutively numbered envelopes, which were opened just before the first injection.

During the preoperative phase, the patients were carefully evaluated to see if they were suitable. In the first visit the clinicians collected patients’ personal data, medical history and dental information.

Photographs of the involved sites were taken. Full mouth supragingival and subgingival instrumentation were performed under local anaesthesia (2% lidocaine hydrochloride with adrenaline 1:80,000), using an ultrasonic instrument (Electro Medical Systems EMS, Nyon, Switzerland) with dedicated tips (Piezon A, P, PS, EMS), and periodontal Gracey’s Curettes (Hu Friedy Co., Ltd., Chicago, IL, USA) if needed. Patients received instructions for maintaining proper oral hygiene. The linear distance from the contact point to the interproximal bone crest was then assessed at the sites of papillary defect, on periapical radiographs obtained using the parallel technique, with customized stents. Only the sites with a distance ≤ 7 mm were eligible for inclusion.

The degree of papillary deficiency, according to the Nordland and Tarnow classification, as well as plaque and gingival indices, were reassessed for eligibility after 4 weeks. An X-ray and a photo were also taken. Only patients with one or more deficient papillary sites that met the inclusion criteria were then recalled.

Before proceeding to injection, the patient allocation was revealed to the clinician. The injection phase always started with the local anaesthesia, using an infiltration technique in the labial vestibule (Lignocaine Hydrochloride 21.3 mg, Adrenaline 1:200,000). The defective papilla was injected with 0.2 mL of 0.8% hyaluronic acid gel (Gengigel^®^, Ricerfarma s.r.l., Milano, Italy) or with 0.2 mL of 0.8% HA gel followed by 0.2 mL PRGF (two consecutive injections). Each injection was performed by a 30-gauge disposable insulin syringe (BD Glide^TM^, Becton, Dickinson and Company, Haryana, India). The needle was inserted 2–3 mm apical to the tip of the interdental papilla and oriented coronally with 45° angulation with respect to the tooth’s axis, and the bevel was directed apically. Then, the papilla was gently massaged for 1 min with digital pressure in an incisal direction using a gauze.

The plasma rich in growth factors (PRGF) was prepared following the manufacturer’s protocol [24]. Nine milliliters of blood was obtained from all the patients of the test group and collected into tubes containing an anticoagulant (3.8% sodium citrate solution). The tubes were centrifuged at 580 g for 8 min at room temperature using an Endoret System centrifuge (Biotechnology Institute BTI S.L. Miñano Álava Spain). At the end of the centrifugation, erhythrocytes at the bottom of the tube and the buffy coat layer in the middle were discarded. The whole plasma above the buffy coat was collected after centrifugation, avoiding the leukocytes layer, using the closed Endoret-PRGF system. The plasma column was separated into two fractions: PRGF just above the buffy coat layer, and plasma poor in growth factors (PPGF) at the top of the tube. The top 2 mL of the plasma was labeled fraction 1 (F1 or plasma poor in growth factors) and the bottom 2 mL fraction 2 (F2 or PRGF). F2 was collected and activated with 0.2 mL of 10% calcium chloride and placed into an insulin syringe for injection into the papilla.

Patients were recalled 12 weeks after the first injection and clinical measurement of black triangles and clinical photographs were taken again.

Post-injection instructions were provided that recommended not performing mechanical plaque control in the area for 24 h and using mouthwashes (0.2% chlorhexidine digluconate (Hexidine, ICPA Health Products Ltd., Mumbai, India) twice daily. After the first 24 h, the use of a soft toothbrush with mouthwash was indicated.

### 2.4. Outcome Variables

All these measurements were taken at baseline, and at each follow-up:

Papillary Width (the black triangle base: the horizontal distance between adjacent teeth at the crestal level of the interdental papilla).

Papillary Deficient Height (the black triangle height: vertical distance from the interdental contact point to the crest of the interdental papilla.

Deficient Area (the black triangle area was estimated from the two previous measurements: Area = 1/2 × base × height).

Deficient Volume (this was calculated from the previous measurements, considering the papillary depth in the bucco-palatal dimension resulting from the addition silicone impressions (Aquasil Ultra Soft Putty, Dentsply Sirona, Charlotte, NC, USA) taken at each follow-up, measured using a verniers caliper with a 0.001 mm precision: Volume = 1/3 × base × height × depth).

**Figure 1:**
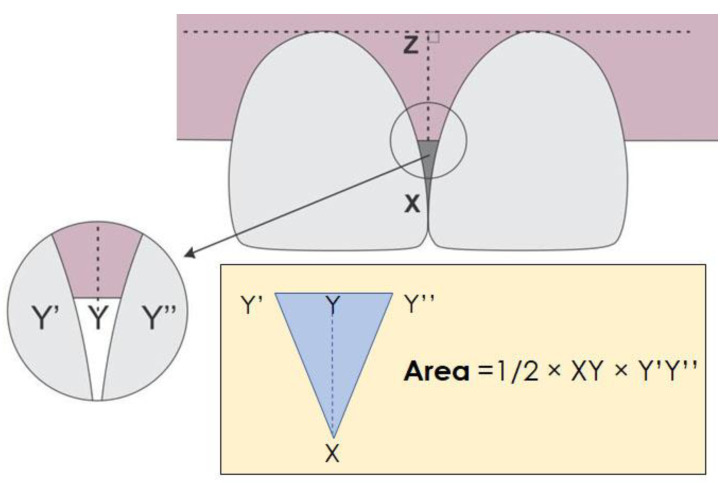
Schematic representation of the reference points. Point X = Interdental Contact Point; Point Y = Crest of the Interdental Papilla; Point Z = Point perpendicular to the imaginary line joining the facial CEJ of adjacent teeth; Y’Y” = Papillary Width; XY = Papillary Deficient Height; YZ = Papillary Height; XZ = Expected Papillary Height.

Figure 1 schematically illustrates the reference points that were used to take measurements.

The measurements were based on photographs that were obtained with a digital camera (Nikon D5300 DSLR, Nikon Corporation, Tokyo, Japan), uploaded to a PC, and analyzed using a digital image processing free software (ImageJ, NIH, USA, https://imagej.net/ij/index.html (accessed on 15 June 2022)). The calibration was performed with a 10 mm UNC-15 periodontal probe.

Due to the treatment performed, neither patients nor operators could be blinded to the group allocation. All clinical and photographic measurements were taken by an independent investigator who was not involved in patients’ treatment, was blinded to the group assignment, and was experienced in both the assessment of periodontal parameters and the usage of the image analysis software for measuring distances on clinical and radiographic images.

### 2.5. Statistical Analysis

The data collected postoperatively at baseline, 3 weeks, 6 weeks, 12 weeks were tabulated and analysed statistically. The site (papilla) was the unit of analysis. SPSS software was used for the statistical analysis (version 20, SPSS, Inc., Chicago, IL, USA). For normally distributed quantitative data, the statistical tests used were paired *t*-test for within-group comparison and unpaired *t*-test for between-group comparison (control versus test groups). Normality of the distributions was estimated using the D’Agostino and Pearsons omnibus normality test. The Levene test for equality of variance was used to check if the variances for Deficient Area and Volume changes were equal. A *p*-value of 0.05 was the significance level.

## 3. Results

**Figure 2:**
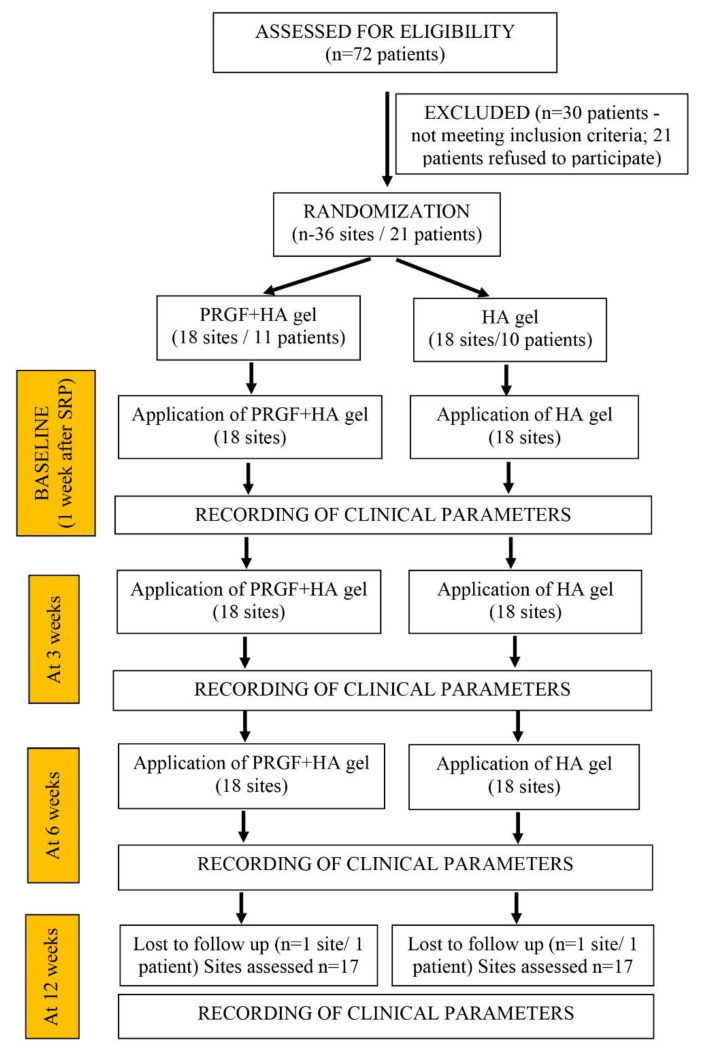
Consort flowchart for the patient recruitment, n = number of sites/patients.

A total of 72 patients were screened as potentially eligible. Thirty patients did not meet the inclusion criteria, and 21 subjects refused to take part in the trial. Therefore, 21 patients (10 males and 11 females) finally participated in the trial. Figure 2 shows a flowchart of the selection process, treatment and follow-up of patients.

The main features of the included subjects in the two groups, regarding clinical periodontal parameters, are reported in Table 1.

The participants age range was 18–45 years. All patients had Class I or Class II interdental papillary loss in the maxillary region. Two patients (one per each group) dropped out in the middle of the study. Finally, 19 patients (10 males, 9 females), with 34 sites (4 patients contributed with 1 site and 15 with 2 sites each) were analysed. In patients contributing with two sites, one site received the test treatment and the other the control one.

**Figure 3:**
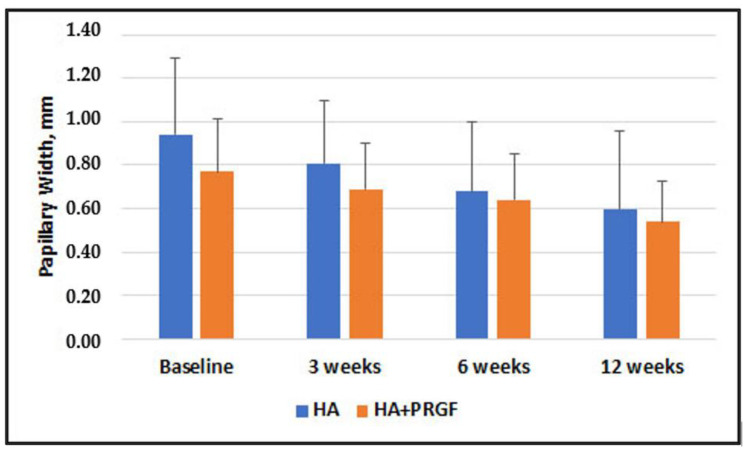
Comparison of Papillary Width in Group HA alone and Group HA + PRGF at baseline, 3 weeks, 6 weeks. No statistically significant improvement was observed in Papillary Width in Group HA + PRGF respect to Group HA alone at baseline, 3 weeks, 6 weeks, 12 weeks.

**Figure 4:**
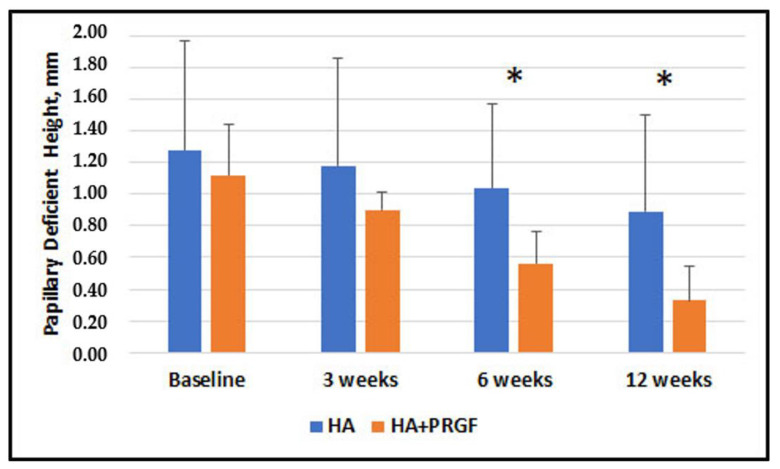
Comparison of Papillary Deficient Height (PDH) in Group HA alone and Group HA + PRGF at baseline, 3 weeks, 6 weeks. No statistically significant improvement was observed in papillary height in Group HA + PRGF respect to Group HA alone at baseline and 3 weeks. There was statistically significant difference from baseline to 6 weeks and 12 weeks. The asterisks * indicate significant between-group difference.

**Figure 5:**
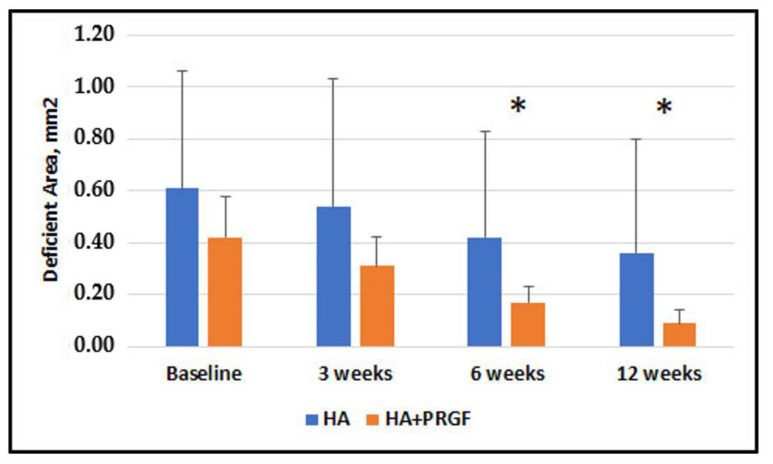
Comparison of Deficient Area (DA) in Group HA alone and Group HA + PRGF at baseline, 3 weeks, 6 weeks and 12 weeks. Statistically significant improvement was observed in Deficient Area (DA) in Group HA + PRGF as compared to Group HA alone at 6 weeks and 12 weeks but not during other time frames. The asterisks * indicate significant between-group difference.

**Figure 6:**
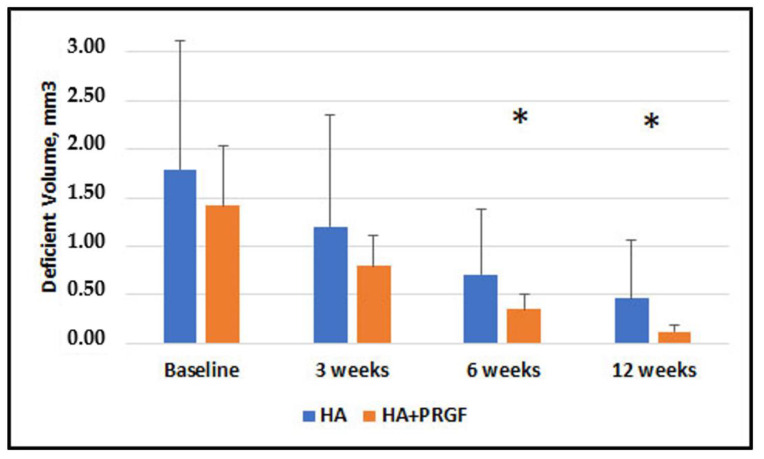
Comparison of Deficient Volume (DV) in Group HA alone and Group HA + PRGF at baseline, 3 weeks, 6 weeks and 12 weeks. Statistically significant improvement was observed in Deficient Volume (DV) in Group HA + PRGF respect to Group HA alone at 3 weeks, 6 weeks and 12 weeks. The asterisks * show significance in the difference between groups.

Figure 3, Figure 4, Figure 5 and Figure 6 present the results of the measurements of the outcome variables in the two groups up to 12 weeks.

Supplementary Tables S1 and S2 show mean values and standard deviations for the two groups, at baseline, 3, 6 and 12 weeks for Papillary Width (mm), Papillary Deficient Height (mm), Deficient Area (mm^2^) and Volume (mm^3^).

Table 2 shows the descriptive statistics of inter-group comparisons for the outcome variables assessed. No statistically significant between-group difference was observed at baseline and 3 weeks. No significant difference was found in Papillary Width at 6 and 12 weeks (*p* = 0.63 and *p* = 0.59, respectively). There was a statistically significant difference in Papillary Deficient Height, Deficient Area, and Deficient Volume between the groups at both 6 and 12 weeks.

Intragroup comparisons of Papillary Width, Papillary Deficient Height, Deficient Area, and Deficient Volume at different times are shown in Supplementary Tables S3–S6. The results at 3, 6, and 12 weeks as compared to baseline and between 3 weeks and 6, 12 weeks showed significant differences in all cases (*p* < 0.05).

Table 3 and Table 4 show the mean percentage change (reduction) in Deficient Area and Deficient Volume, respectively, at 3, 6 and 12 weeks. A statistically significant difference between groups was found in the percentage change of Deficient Area (*p* < 0.001) at 6 and 12 weeks, but no significant difference was observed at 3 weeks. All differences in Deficient Volume changes were significant.

Supplementary Tables S7 and S8 show more detailed statistical results regarding the percentage changes in Deficient Area and Deficient Volume, respectively.

**Figure 7:**
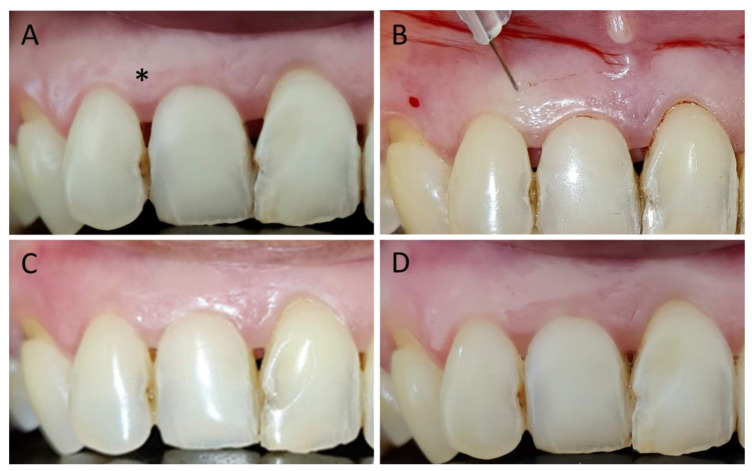
Hyaluronic acid gel injection. The asterisk indicates the treated papilla. (**A**) Interdental papillary loss at baseline. (**B**) Application of HA. (**C**) Follow up at 3 weeks. (**D**) Follow up at 12 weeks.

Figure 7 describes a clinical case of hyaluronic acid gel injection (control group) and the follow up at different times.

**Figure 8:**
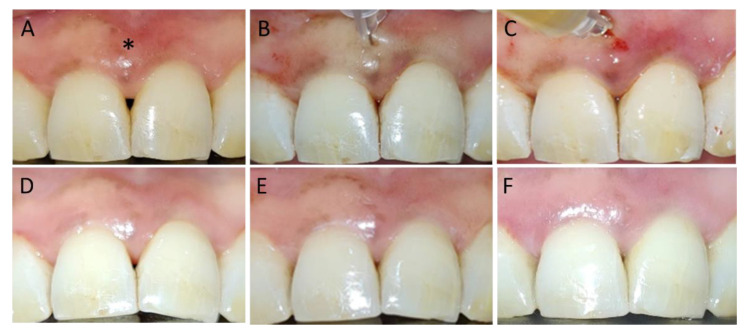
Hyaluronic acid gel injection with PRGF. The asterisk indicates the treated papilla. (**A**) Interdental papillary loss at baseline. (**B**) Application of HA. (**C**) Application of PRGF. (**D**) Follow up at 3 weeks. (**E**) Follow up at 6 weeks. (**F**) Follow up at 12 weeks.

Figure 8 describes a clinical case of hyaluronic acid gel injection plus PRGF (test group) and the follow up at different times.

## 4. Discussion

This clinical study was conducted to analyze and compare the relative effectiveness of two treatment modalities in the management of lost interdental papilla in the anterior maxilla, using injectable HA with and without PRGF.

The results showed absence of statistically significant difference between groups in the mean PDH, DA and DV at baseline and 3 weeks. However, there was a statistically significant difference in PDH, DA, DV at 6- and 12-week follow-ups, favoring the group in which PRGF was additionally used. An intragroup comparison of PW, PDH, DA, and DV shows a statistically significant difference at all study timelines, suggesting that both treatments may produce beneficial results in reducing the black triangle.

The results of this randomized trial regarding the effect of PRGF in adjunct to HA compared to HA alone cannot be directly compared with other studies, because no published study has investigated injectable HA versus HA plus PRGF for the regeneration of anterior interdental papilla. Conversely, several studies reported beneficial results of HA injections for the treatment of papillary loss. Different HA concentrations, injection protocols and follow-up duration were reported, with variable results. Singh & Vandana. evaluated three HA concentrations (1%, 2%, and 5%) [29]. Using 1% HA solution (the closest to the one used in the present study), they found a reduction in the black triangle area by 17%, 18.8% and 14.2% at 1, 3, and 6 months, respectively. In our study, the area reduction in the HA group at 6 weeks was 20.2%, similar to that of Singh and Vandana. Interestingly, they found that the highest HA concentration tested (5%) produced the best result, showing a Deficient Area reduction of 41%, 42.9%, and 39.8% at 1, 3 and 6 months, respectively [29]. The latter results are inferior to those found in the present study at any follow-up in the HA + PRGF group.

Ni et al. evaluated injections of 16 mg/mL HA (corresponding to 1.6%) against physiological saline solution in the reconstruction of deficient gingival papillae [11]. The multiple injection protocol was similar to ours (baseline, 3 and 6 weeks later), and the follow-up was at 6 and 12 months. HA significantly enhanced the defective gingival papillae compared to physiological saline solution. The Deficient Area reduction was 13.6% and 23.6% at 6 and 12 months, respectively, which is slightly worse than ours, in spite of a double HA concentration used. It is possible that in the Ni et al. study [11], the larger baseline Deficient Area with respect to our study (1.9 vs. 0.61 mm^2^) reflected a reduced regenerative potential.

Pitale et al. assessed linear changes of black triangle height and papilla width after a single injection of 2% HA in lost interdental papillae [12]. The black triangle height reduction in the Pitale et al. study was better than in our HA group, possibly due to the higher HA concentration used. Notably, the addition of PRGF in the present study produced a marked improvement in deficient height reduction, even superior to that observed by Pitale et al. [12].

In our study, we carried out a photographic measurement of clinical parameters rather than conventional clinical measurement. Alhabashneh et al. [13] also used digital photographs to evaluate the esthetic of deficient papillary sites compared to baseline. Digital photographs were then processed through software ImageJ^®^ analysis and calibrated using pixels, as we have done in our study. One of the major advantages of photographic evaluation is the expansion of the linear scale of measurement to 100th of a millimeter, which allows a greater precision in comparison to conventional UNC 15 periodontal probe, which has 1 mm markings. Alternatively, a vernier caliper may be used. However, the advantage of repeatability of digital measurement on a photograph by-far outweighs the advantages of physical measurement [10,14]. On the other hand, the photographic assessment could have potential limits that can be viewed as an instrument bias. In fact, the sensor sensibility, exposure settings, and axis alteration when taking the pictures could represent a source of deviation. Previous evidence supports the fact that PRGF represents a source of growth factors involved in tissue regeneration, which may stimulate soft tissues, promoting epithelization, cell adhesion and migration [20,21,22,26]. In addition to a broad literature supporting the beneficial effects of PRGF in both topical and injectable forms, the simplicity of the protocol, the advantageous cost/effectiveness and the ready availability of the system oriented our choice towards this product.

The observed greater change in the PRGF group at 6 and 12 weeks may be hypothesized to be related to the fact that PRGF-gel is similar to the native skin environment and resists the physiological forces of tension, pressure and tissue activity, ensuring a long-lasting support, as reported in the dermatological field [30,31]. Hence, we can deduce that it would have similar effects in the gingiva as well. Furthermore, previous studies suggested that PRGF has synergistic effects when used along with HA that would aid in the augmentation of interdental papilla [25,26,30].

Anitua et al. [26] tried to assess if PRGF-Endoret^®^ could promote the migration of tendon cells and synovial fibroblasts, and evaluated whether this autologous technology with hyaluronic acid (HA) improves the potential of biomaterials to stimulate the mobility of both types of fibroblasts. They observed that PRGF was able to strongly stimulate both tendon fibroblasts and synovial fibroblasts. Hence, in our study, we tried to achieve a greater stability of HA and increase the size of the deficient papilla by combining the two biological substances.

Among the limitations of this study is the mixed study design, which was dictated by the availability of cases. With split-mouth cases, which represented the majority (88% of cases, or nearly 80% of patients), there is the advantage of a reduced “background noise”, which allows us to increase the power of the test (reducing the need for a larger sample size). On the other hand, the risk exists that differences between groups might be underestimated, due to a possible carry-across effect of the PRGF between test and control sites. Furthermore, the follow up time of 12 weeks may appear short as compared to other studies in the literature [11,12,13,29,32]. Considering the biological healing period needed for this kind of gingival defect, further studies with longer follow-up could produce more interesting findings regarding the effectiveness and clinical advantages resulting from the combination of injectable HA with PRGF. The application of the biomaterials was performed thrice within the given span of 12 weeks at the interval of 21 days, similar to previous studies that used HA alone [5,8,11,12]. A longer follow up time (e.g., 6 months to 1 year) could have allowed us to evaluate the stability of the results and determine if the benefits observed with the adjunct of PRGF are maintained over time. In addition, different injection protocols could be tested, such as increasing the number of applications to five or six times, in order to determine if it is possible to achieve better and durable results. Patients reported no pain or discomfort throughout the duration of the study; however, such outcomes were not systematically assessed and analysed. We plan to evaluate postoperative pain and discomfort as well as patient satisfaction in future studies, in order to enhance our ability to provide better care and treatment options to our patients. Additionally, gingival tissue thickness was not assessed in the present study, which might be evaluated as a factor possibly affecting the results. In fact, gingival tissue thickness or phenotype is essential for complete root coverage and the stability of the clinical outcome [33,34].

An additional limitation in the present study could be the lack of taking the shape of the tooth into consideration as well as the periodontal phenotype. The tooth shape determines the gingival scallop degree. Teeth with a triangular shape form a pronounced scallop and may influence the occurrence of “black triangles,” especially in the presence of a thin biotype. Moreover, the roots of triangular teeth tend to diverge, with greater interproximal bone thickness, resulting in less vertical bone loss with respect to square teeth [35]. However, square teeth allow for better interproximal papilla maintenance, because the interproximal distance between the contact point and the osseous crest is smaller than triangular teeth.

The strengths of our study are that, as far as we know, it is the first randomized trial that compares HA versus HA + PRGF injections as a papilla reconstruction treatment. Recent systematic reviews highlighted that studies addressing the benefits of HA for papillary reconstruction have a low level of evidence, with the majority of studies represented by case series [3,12]. Before our study, only three randomized studies were conducted, where HA was compared to saline [11,15,36], yet so far, no published comparative studies have used any other injectable filler in addition to HA. The findings of the present clinical trial confirm that autologous platelet concentrates may provide an excellent adjunctive effect for papilla reconstruction, as was widely shown for periodontal regeneration [18,37,38,39,40,41].

Furthermore, in our study, we have also tried to measure the papillary depth by using a vernier caliper on the impressions made by using additional silicone. This unique method proposed in our study provides a three-dimensional picture of the deficient interdental papilla and the amount of volume gained throughout the study.

## 5. Conclusions

The black triangle that results due to interdental papilla loss is among the most challenging dilemmas in the field of dentistry as it predisposes the patient to phonetic, functional and esthetic problems. The use of PRGF and HA for the minimally invasive management of papillary loss is associated with various beneficial outcomes, including rapid and effective healing, and it holds promise for further investigations. The present study demonstrated that there was additional benefit in the clinical parameters with the combined use of HA and PRGF. Further long-term clinical trials investigating the full potential of such a combination are still needed to confirm that PRGF is additionally beneficial in increasing volume and enhancing the hygroscopic properties of HA when used together. Furthermore, additional evidence in the literature is needed to standardize the amount, duration and frequency of injecting PRGF when used in combination with HA to provide the best desired results.

## Figures and Tables

**Table 1:** Main clinical/periodontal parameters of the patients of the two groups at baseline. Data are reported as mean value ± standard deviation (SD).

Patient Characteristics	HA Group	HA + PRGF Group
No. of sites/patients	18/10	18/11
Age, years	34.63 ± 5.22	38.71 ± 8.4
Probing Depth, mm	2.82 ± 1.1	2.54 + 0.71
Clinical Attachment Level, mm	3.24 ± 0.91	2.92 ± 0.97
Gingival Recession, mm	0.7 ± 0.2	0.8 ± 0.3
Papillary Width, mm	0.94 ± 0.35	0.77 ± 0.24
Papillary Deficient Height, mm	1.28 ± 0.69	1.12 ± 0.32
Deficient Area, mm^2^	0.61 + 0.45	0.42 + 0.16
Deficient Volume, mm^3^	1.79 ± 1.32	1.42 ± 0.62
Contact point to Bone Crest, mm	5.26 ± 1.2	5.92 ± 0.72

**Table 2:** Inter-group comparison of Papillary Width, Papillary Deficient Height, Deficient Area and Deficient Volume at baseline, 3, 6, and 12 weeks, using unpaired Student’s *t* Test.

	Baseline			3 Weeks			6 Weeks			12 Weeks		
	Mean Difference (SE)	95% CI of the Difference	*p*-Value	Mean Difference (SE)	95% CI of the Difference	*p*-Value	Mean Difference (SE)	95% CI of the Difference	*p*-Value	Mean Difference (SE)	95% CI of the Difference	*p*-Value
Papillary Width, mm	0.18 (0.10)	−0.02, 0.38	0.08	0.12 (0.08)	−0.05, 0.29	0.16	0.04 (0.09)	−0.14, 0.23	0.63	0.05 (0.10)	−0.15, 0.25	0.59
Papillary Deficient Height, mm	0.16 (0.18)	−0.20, 0.53	0.37	0.28 (0.16)	−0.05, 0.61	0.09	0.49 (0.13)	0.22, 0.76	0.001 *	0.56 (0.16)	0.24, 0.88	0.001 *
Deficient Area, mm^2^	0.19 (0.11)	−0.04, 0.42	0.11	0.22 (0.12)	−0.02, 0.47	0.07	0.24 (0.10)	0.04, 0.44	0.019 *	0.27 (0.11)	0.05, 0.49	0.017 *
Deficient Volume, mm^3^	0.37 (0.34)	−0.33, 1.07	0.29	0.41 (0.28)	−0.16, 0.98	0.16	0.36 (0.16)	0.03, 0.69	0.032 *	0.33 (0.15)	0.03, 0.64	0.032 *

SE = standard error of the difference; CI = confidence interval; * = significant difference at *p* < 0.05.

**Table 3:** Descriptive statistics of percentage change in Deficient Area between Group HA alone and Group HA + PRGF.

Time and Group		N	Mean, %	Std. Deviation, %	*p*-Value
3 Weeks	HA	18	12.07	8.96	0.340
	HA + PRGF	18	15.94	14.41	
6 Weeks	HA	18	20.24	10.80	0.000
	HA + PRGF	18	49.30	18.01	
12 Weeks	HA	17	57.62	21.78	0.006
	HA + PRGF	17	77.42	16.70	

**Table 4:** Descriptive statistics of percentage change in Deficient Volume between Group HA alone and Group HA + PRGF.

Time and Group		N	Mean, %	Std. Deviation, %	*p*-Value
3 Weeks	HA	18	23.60	15.87	0.000
	HA + PRGF	18	42.28	11.91	
6 Weeks	HA	18	56.03	20.55	0.006
	HA + PRGF	18	73.14	13.83	
12 Weeks	HA	17	81.42	17.20	0.033
	HA + PRGF	17	91.19	5.70	

## Data Availability

The authors are available to share the data upon request.

## Supplementary Materials

The following supporting information can be downloaded at: https://www.mdpi.com/article/10.3390/jfb14020114/s1, Table S1. Descriptive statistics of Papillary Width, Papillary Deficient Height, Deficient Area, Deficient Volume values of Group Hyaluronic acid alone (HA) at baseline, 3 weeks, 6 weeks, 12 weeks. Table S2. Descriptive statistics of Papillary Width, papillary Deficient Height, Deficient Area, Deficient Volume values of Group Hyaluronic acid (HA) + PRGF at baseline, 3 weeks, 6 weeks, 12 weeks. Table S3. Intragroup comparisons of Papillary Width (mm) at baseline, 3, 6 and 12 weeks for both groups. Table S4. Intragroup comparisons of Papillary Deficient Height (mm) at baseline, 3, 6 and 12 weeks for both groups. Table S5. Intragroup comparisons of Deficient Area (mm2) at baseline, 3 weeks, 6 weeks and 12 weeks for Group HA alone. Table S6. Intragroup comparisons of Deficient Volume (mm3) at baseline, 3 weeks, 6 weeks and 12 weeks for Group HA alone. Table S8. Intergroup comparison of percentage change in Deficient Volume between Group HA alone and Group HA+ PRGF.
