# Supplementary material for: Effectiveness of Hyaluronic Acid Gel Injection with and without PRGF for Management of Interdental Papillary Loss: A Randomized Clinical Trial
Source: J Funct Biomater. 2023 Feb 18;14(2):114. doi: 10.3390/jfb14020114 (PMC9967875; doi:10.3390/jfb14020114)
Supplement: Supplementary file 1 [file jfb-14-00114-s001.zip › jfb-2194442-supplementary.pdf]

Supplementary Table S1. Descriptive statistics of Papillary Width, Papillary Deficient Height, Deficient Area, Deficient Volume values of Group Hyaluronic acid alone (HA) at baseline, 3 weeks, 6 weeks, 12 weeks

	HA							
	Baseline (n=18)		3 Weeks (n=18)		6 Weeks (n=18)		12 Weeks (n=17)	
	Mean	Standard Deviation	Mean	Standard Deviation	Mean	Standard Deviation	Mean	Standard Deviation
Papillary Width, mm	0.94	0.35	0.81	0.29	0.68	0.32	0.60	0.36
Papillary Deficient Height, mm	1.28	0.69	1.18	0.68	1.04	0.53	0.89	0.61
Deficient Area, mm^2	0.61	0.45	0.54	0.49	0.42	0.41	0.36	0.44
Deficient Volume, mm^3	1.79	1.32	1.20	1.15	0.71	0.67	0.46	0.61

Supplementary Table S2. Descriptive statistics of Papillary Width, papillary Deficient Height, Deficient Area, Deficient Volume values of Group Hyaluronic acid (HA) + PRGF at baseline, 3 weeks, 6 weeks, 12 weeks

	HA + PRGF							
	Baseline (n=18)		3 Weeks (n=18)		6 Weeks (n=18)		12 Weeks (n=17)	
	Mean	Standard Deviation	Mean	Standard Deviation	Mean	Standard Deviation	Mean	Standard Deviation
Papillary Width, mm	0.77	0.24	0.69	0.21	0.64	0.21	0.54	0.19
Papillary Deficient height, mm	1.12	0.32	0.90	0.11	0.56	0.20	0.33	0.21
Deficient Area, mm^2	0.42	0.16	0.31	0.11	0.17	0.06	0.09	0.05
Deficient Volume, mm^3	1.42	0.62	0.80	0.31	0.35	0.15	0.12	0.07

Supplementary Table S3. Intragroup comparisons of Papillary Width (mm) at baseline, 3, 6 and 12 weeks for both groups.

Paired Samples Test[a]									
		Paired Differences					T	Df	p-value
		Mean	Standard Deviation	Standard Error Mean	95% Confidence Interval of the Difference				
					Lower	Upper			
HA	Baseline - 3 Weeks	0.14	0.22	0.05	0.03	0.25	2.60	17.00	0.019
HA+PRGF		0.08	0.10	0.02	0.03	0.13	3.49	17.00	0.003
HA	Baseline - 6 Weeks	0.26	0.30	0.07	0.11	0.41	3.73	17.00	0.002
HA+PRGF		0.13	0.12	0.03	0.07	0.19	4.60	17.00	< 0.001
HA	Baseline - 12 Weeks	0.34	0.36	0.09	0.15	0.52	3.89	16.00	0.001
HA+PRGF		0.23	0.17	0.04	0.14	0.32	5.59	16.00	< 0.001
HA	3 Weeks - 6 Weeks	0.13	0.10	0.02	0.08	0.17	5.38	17.00	< 0.001
HA+PRGF		0.05	0.06	0.01	0.02	0.08	3.25	17.00	0.005
HA	3 Weeks - 12 Weeks	0.20	0.15	0.04	0.12	0.28	5.41	16.00	< 0.001
HA+PRGF		0.15	0.12	0.03	0.09	0.21	5.34	16.00	< 0.001
HA	6 Weeks - 12 Weeks	0.08	0.06	0.01	0.04	0.11	5.13	16.00	< 0.001
HA+PRGF		0.11	0.07	0.02	0.07	0.15	6.14	16.00	< 0.001

Supplementary Table S4. Intragroup comparisons of Papillary Deficient Height (mm) at baseline, 3, 6 and 12 weeks for both groups.

Paired Samples Test[a]									
		Paired Differences					t	df	p-value
		Mean	Standard Deviation	Standard Error Mean	95% Confidence Interval of the Difference				
					Lower	Upper			
HA	Baseline - 3 Weeks	0.10	0.06	0.01	0.07	0.13	7.14	17.00	< 0.001
HA+PRGF		0.22	0.30	0.07	0.07	0.37	3.06	17.00	0.007
HA	Baseline - 6 Weeks	0.24	0.17	0.04	0.15	0.32	6.08	17.00	< 0.001
HA+PRGF		0.56	0.30	0.07	0.41	0.71	7.95	17.00	< 0.001
HA	Baseline - 12 Weeks	0.41	0.23	0.06	0.29	0.52	7.35	16.00	< 0.001
HA+PRGF		0.80	0.30	0.07	0.65	0.96	10.99	16.00	< 0.001
HA	3 Weeks - 6 Weeks	0.14	0.17	0.04	0.05	0.22	3.44	17.00	0.003
HA+PRGF		0.34	0.18	0.04	0.25	0.44	7.89	17.00	< 0.001
HA	3 Weeks - 12 Weeks	0.31	0.21	0.05	0.20	0.41	6.05	16.00	< 0.001
HA+PRGF		0.58	0.22	0.05	0.47	0.69	10.82	16.00	< 0.001
HA	6 Weeks - 12 Weeks	0.17	0.20	0.05	0.06	0.27	3.45	16.00	0.003
HA+PRGF		0.22	0.10	0.02	0.17	0.28	8.97	16.00	< 0.001

Supplementary Table S5. Intragroup comparisons of Deficient Area (mm^2) at baseline, 3 weeks, 6 weeks and 12 weeks for Group HA alone

Paired Samples Test[a]									
		Paired Differences					t	df	p- value
		Mean	Std. Deviation	Std. Error Mean	95% Confidence Interval of the Difference				
					Lower	Upper			
HA	Baseline - 3 Weeks	0.07	0.13	0.03	0.01	0.14	2.39	17.00	0.028
HA+PRGF		0.11	0.12	0.03	0.05	0.17	3.97	17.00	0.001
HA	Baseline - 6 Weeks	0.19	0.15	0.04	0.12	0.27	5.41	17.00	< 0.001
HA+PRGF		0.25	0.14	0.03	0.18	0.32	7.40	17.00	< 0.001
HA	Baseline - 12 Weeks	0.25	0.19	0.05	0.15	0.35	5.41	16.00	< 0.001
HA+PRGF		0.34	0.16	0.04	0.26	0.42	8.94	16.00	< 0.001
HA	3 Weeks - 6 Weeks	0.12	0.09	0.02	0.07	0.17	5.44	17.00	< 0.001
HA+PRGF		0.14	0.09	0.02	0.09	0.18	6.52	17.00	< 0.001
HA	3 Weeks - 12 Weeks	0.18	0.11	0.03	0.12	0.24	6.66	16.00	< 0.001
HA+PRGF		0.23	0.12	0.03	0.17	0.29	8.09	16.00	< 0.001
HA	6 Weeks - 12 Weeks	0.06	0.05	0.01	0.03	0.09	4.85	16.00	< 0.001
HA+PRGF		0.09	0.04	0.01	0.07	0.11	9.67	16.00	< 0.001

Supplementary Table S6. Intragroup comparisons of Deficient Volume (mm^3) at baseline, 3 weeks, 6 weeks and 12 weeks for Group HA alone.

Paired Samples Test[a]										
		Paired Differences					t	df	p-value
		Mean	Std. Deviation	Std. Error Mean	95% Confidence Interval of the Difference				
					Lower	Upper			
HA	Baseline - 3 Weeks	0.59	0.49	0.11	0.35	0.83	5.14	17.00	< 0.001
HA+PRGF		0.63	0.39	0.09	0.43	0.82	6.84	17.00	<0.001
HA	Baseline - 6 Weeks	1.08	0.74	0.18	0.71	1.45	6.15	17.00	< 0.001
HA+PRGF		1.07	0.55	0.13	0.80	1.35	8.24	17.00	0.000
HA	Baseline - 12 Weeks	1.33	0.86	0.21	0.89	1.78	6.41	16.00	< 0.001
HA+PRGF		1.33	0.60	0.15	1.02	1.65	9.10	16.00	0.000
HA	3 Weeks - 6 Weeks	0.49	0.52	0.12	0.23	0.75	3.98	17.00	0.001
HA+PRGF		0.44	0.21	0.05	0.34	0.55	8.91	17.00	0.000
HA	3 Weeks - 12 Weeks	0.75	0.58	0.14	0.45	1.05	5.29	16.00	< 0.001
HA+PRGF		0.69	0.28	0.07	0.55	0.84	10.24	16.00	0.000
HA	6 Weeks - 12 Weeks	0.26	0.18	0.04	0.17	0.35	6.05	16.00	< 0.001
HA+PRGF		0.24	0.10	0.02	0.18	0.29	9.78	16.00	0.000

Supplementary Table S7. Intergroup Comparisons of percentage change in Deficient Area between Group HA alone and Group HA+ PRGF.

	Levene's Test for Equality of Variances	t-test for Equality of Means						
	F	t	df	p value	Mean Difference	Std. Error Difference	95% Confidence Interval of the Difference	
							Lower	Upper
3 Weeks	0.03	-0.97	34.00	0.340	-3.87	4.00	-12.00	4.26
6 Weeks	3.47	-5.87	34.00	0.000	-29.06	4.95	-39.12	-19.00
12 Weeks	0.52	-2.97	32.00	0.006	-19.80	6.66	-33.36	-6.24

Supplementary Table S8. Intergroup comparison of percentage change in Deficient Volume between Group HA alone and Group HA+ PRGF.

	Levene's Test for Equality of Variances	t-test for Equality of Means						
	F	t	df	p value	Mean Difference	Std. Error Difference	95% Confidence Interval of the Difference	
							Lower	Upper
3 Weeks	0.66	-4.00	34.00	0.000	-18.69	4.68	-28.19	-9.18
6 Weeks	2.49	-2.93	34.00	0.006	-17.11	5.84	-28.97	-5.24
12 Weeks	8.96	-2.22	32.00	0.033	-9.77	4.39	-18.72	-0.82
